# Anticariogenic Potential of Coffee in Adolescents: A Retrospective Exploratory Cohort Study

**DOI:** 10.3390/children13030378

**Published:** 2026-03-07

**Authors:** Murad Alrashdi

**Affiliations:** Department of Orthodontics and Pediatric Dentistry, College of Dentistry, Qassim University, Buraydah 52571, Saudi Arabia; mu.alrashidi@qu.edu.sa

**Keywords:** Coffee, dental caries, *Streptococcus mutans*, anticariogenic

## Abstract

**Background**: Dental caries remains one of the most prevalent childhood diseases worldwide, with *Streptococcus mutans* playing a major etiological role. Coffee contains bioactive compounds, including chlorogenic acid and trigonelline, which have demonstrated antimicrobial and anticariogenic properties. Limited evidence exists on the preventive potential of coffee in Saudi adolescents. This study was designed to assess the benefits of coffee consumption in reducing dental caries among adolescents aged 12 to 16 years. **Methods**: A retrospective cohort study was conducted among 375 participants aged 12–16 years in Saudi Arabia. Participants were recruited from dental records and allocated into two cohorts: (1) consuming coffee at least twice weekly, subcategorized as sweetened versus unsweetened coffee consumers, and (2) non-coffee consumers. Clinical assessment included the Decayed, Missing, and Filled Teeth (DMFT) index, which was assessed at variable follow-up times up to 5 years. Caries increment (ΔDMFT) was compared using independent samples t-tests and one-way ANOVA with Tukey’s post hoc tests. Multivariable linear regression adjusted for tooth brushing frequency, dietary habits, and oral hygiene status was performed. **Results**: Coffee consumers had significantly lower caries increment than non-consumers (0.78 ± 0.65 vs. 1.34 ± 0.88; mean difference −0.56; 95% CI −0.71 to −0.41; *p* < 0.001; Hedges’ g = −0.71). Among coffee consumers, unsweetened coffee was associated with a lower increment than sweetened coffee (0.52 ± 0.48 vs. 1.09 ± 0.71; mean difference −0.57; 95% CI −0.76 to −0.38; *p* < 0.001) and non-consumers (mean difference −0.82; 95% CI −0.98 to −0.66; *p* < 0.001). After adjusting for oral hygiene and dietary factors, the protective associations remained significant for both unsweetened (adjusted mean difference −0.51; 95% CI −0.70 to −0.33; *p* < 0.001) and sweetened coffee (adjusted mean difference −0.22; 95% CI −0.41 to −0.04; *p* = 0.019). **Conclusions**: Within the limitations of this retrospective exploratory design, habitual coffee consumption, particularly that of unsweetened coffee, was associated with lower caries increment. These findings are hypothesis-generating and require confirmation in prospective studies with standardized exposure assessment and biological outcome measures.

## 1. Introduction

Dental caries continues to be one of the most prevalent chronic conditions affecting children and adolescents worldwide. Despite decades of preventive interventions, including fluoridation programs, improved oral hygiene awareness, and routine dental check-ups, the burden of caries remains significant, particularly in developing and middle-income countries. According to the Global Burden of Disease Study (2017), untreated dental caries in permanent teeth was the most common health condition globally, with over 2.3 billion cases reported [1]. Childhood and adolescence represent critical life stages during which oral health behaviors are formed, and the cumulative risk of caries becomes evident. In this context, exploring low-cost preventive measures has become an area of growing importance.

The prevalence of dental caries varies across regions. In Saudi Arabia, national surveys indicate caries prevalence rates of up to 70–90% among adolescents, with DMFT scores often exceeding WHO benchmarks for acceptable oral health [2,3]. Factors contributing to this trend include frequent sugar consumption, limited use of preventive dental services, and cultural dietary practices. The persistence of caries despite widespread knowledge of preventive strategies highlights the need for adjunctive approaches that complement conventional methods. Traditional approaches to caries control have relied heavily on fluoride-based interventions, including fluoridated toothpaste, community water fluoridation, and topical applications, such as varnishes and gels [4,5]. Fluoride strengthens enamel, inhibits bacterial metabolism, and enhances remineralization [6]. Chlorhexidine mouth rinses have also been employed to suppress *S. mutans* levels, particularly in high-risk individuals [7]. While effective, these strategies present limitations. Fluoride use at high levels may increase the risk of dental fluorosis in children, while chlorhexidine is associated with side effects such as tooth staining, altered taste perception, and mucosal irritation [8]. Furthermore, adherence to these regimens may be suboptimal among adolescents, who often prioritize convenience and taste over preventive practices.

Coffee, one of the most widely consumed beverages globally, has attracted increasing interest for its bioactive properties. In Saudi Arabia and many Middle Eastern countries, coffee is not only a dietary component but also a cultural symbol deeply embedded in daily life and social traditions. This widespread acceptance makes it a promising vehicle for preventive health benefits if proven effective. Coffee contains several biologically active compounds, notably chlorogenic acid and trigonelline [9,10]. Each of these has demonstrated potential anticariogenic effects in laboratory and clinical studies. Chlorogenic acid has been shown to inhibit bacterial growth and biofilm formation, particularly against *S. mutans* [11]. It also exhibits antioxidant activity, which may indirectly support oral tissue health. Trigonelline has demonstrated inhibitory effects on microbial enzymatic activity and plaque acidogenicity [12]. Experimental studies have suggested that coffee polyphenols can reduce bacterial colonization, suppress acid production, and inhibit glucosyltransferase, an enzyme critical for *S. mutans* biofilm formation [13]. Although laboratory studies demonstrate antimicrobial activity of coffee polyphenols against *Streptococcus mutans*, clinical evidence evaluating caries outcomes remains limited. In Saudi Arabia, limited data exist on the direct impact of coffee on oral health, despite its widespread consumption from an early age. This study aims to evaluate whether habitual coffee intake confers measurable protective effects against dental caries. We hypothesized that adolescents who consume coffee regularly, particularly unsweetened coffee, would exhibit a lower increment in DMFT over follow-up compared with non-consumers.

## 2. Materials and Methods

### 2.1. Study Design and Setting

This retrospective cohort study was conducted between January 2020 and January 2025. Baseline dental assessments were retrieved from patient records using the Decayed, Missing, and Filled Teeth (DMFT) index. Participants were followed over the five-year study period through existing dental health records to assess changes in caries increment. Each participant had an index date defined as the date of their earliest qualifying baseline examination within the study window. Outcomes were ascertained from routine care records occurring after the index date and up to 5 years post-index when available. Coffee consumption status was also ascertained at both baseline and at the follow-up examination. For participants without sufficient record-based follow-up up to 5 years, an active telephone call to invite patients for a structured interview was implemented. However, all DMFT outcomes were derived exclusively from documented clinical examinations. This study was reported in accordance with the STROBE guidelines for observational studies.

### 2.2. Study Population

The study population comprised adolescents aged 12 to 16 years who had attended Qassim University dental clinic in Saudi Arabia between January 2020 and January 2025. A total of 375 participants were included in the analysis. Eligible participants were identified through clinical records that documented baseline oral health assessments, dietary history, and coffee consumption habits. Participants were categorized into two primary groups based on self-reported dietary history: (1) adolescents who consumed coffee at least twice per week as part of their regular diet, and (2) adolescents who did not consume coffee.

### 2.3. Sample Size and Sampling Technique

The sample size was estimated to ensure adequate statistical power to detect a clinically meaningful difference in caries increment between coffee consumers and non-consumers. An expected standardized mean difference (Hedges’ g) of 0.35 was assumed, corresponding to a medium effect size based on empirical benchmarks derived from meta-analyses in dental research [14]. Using a two-sided significance level (α) of 0.05 and a statistical power (1 − β) of 0.80, the minimum required sample size was calculated to be 172 participants per group. To account for potential loss of records, incomplete data, and subgroup analyses, the target sample size was increased by 10%. This adjustment yielded a total required sample of approximately 375 adolescents. A convenience sampling technique was applied, drawing all eligible charts from the Qassim University dental clinic between January 2020 and January 2025.

### 2.4. Eligibility

#### 2.4.1. Inclusion Criteria

Records were eligible if they pertained to adolescents aged 12–16 years at baseline with care provided at the Qassim University dental clinic and contained: (i) a comprehensive clinical examination with a recorded DMFT score at baseline; (ii) a subsequent routine examination during the study window; and (iii) a documented dietary history specifying coffee consumption frequency (≥2 times/week vs none) and preparation. Only charts with basic oral-hygiene information and general dietary notes sufficient to identify regular intake of sugary snacks/drinks were included.

#### 2.4.2. Exclusion Criteria

Records were excluded if they indicated: (i) systemic conditions or long-term medications known to alter salivary flow or composition, (ii) history of head and neck radiation, (iii) fixed orthodontic appliances in place for a substantial portion of the follow-up, (iv) documented long courses of antibiotics or antimicrobial mouthrinses overlapping outcome assessments, (v) extensive enamel developmental defects or hypoplasia that could confound DMFT scoring, (vi) missing/insufficient data on baseline DMFT, follow-up examination, or coffee exposure, (vii) significant deviation from standard fluoride exposure, such as documented use of high-concentration prescription fluoride toothpaste not related to a specific caries outbreak, or residence in an area with a known non-fluoridated water supply, (viii) a history of fluoride varnish application or sealant placement on any permanent molars within the 12 months preceding baseline, (ix) regular consumption of other polyphenol-rich beverages with known antimicrobial properties such as green tea or black tea, (x) extreme dietary patterns defined as daily consumption of sugar-sweetened beverages (≥2 servings/day) or frequent between-meal sugary snacks (≥3 times/day) and poor oral hygiene (<2 times tooth brushing per day).

#### 2.4.3. Outcome Measures

The primary outcome was dental caries increment. Caries experience over follow-up, quantified as the increment in DMFT between baseline and the most recent routine examination recorded within the study window. DMFT values at both time points were abstracted from clinical records. Lesions were counted at the cavitated level, and “M” and “F” components were attributed to caries when documented as such in the chart. The increment (ΔDMFT) was treated as a continuous variable for group comparisons between coffee consumers and non-consumers. To account for the variability in follow-up duration across participants, all reported caries increment values (ΔDMFTs) were annualized by dividing the absolute increment by individual follow-up time in years. This standardization allows for valid comparisons between participants with different observation periods.

#### 2.4.4. Data Collection

Clinical and dietary data were abstracted retrospectively from routine patient records at the Qassim dental clinic for the period of January 2020–January 2025. Using a structured chart-abstraction form, trained reviewers extracted: (i) baseline and most recent DMFT scores recorded during standard dental examinations; (ii) coffee consumption frequency and habitual sweetening practice from the documented dietary history; and (iii) basic covariates (age, sex, tooth brushing frequency, notes on sugary snacks/drinks, relevant medical history/medications). DMFT values were taken as documented by the attending dentist using WHO cavitated-lesion criteria during routine care. Where charts differentiated in components, D, M, and F counts were captured separately in addition to the total. Details such as daily volume, brew concentration, preparation method (e.g., boiling time), exact sugar quantity, or intake timing relative to meals were not quantified in charts. It is important to note that dietary and oral hygiene information was derived from routine clinical records rather than standardized, validated research instruments. The documentation of coffee intake, sugar use, and dietary habits was based on clinician-recorded notes during routine care and may be subject to recall bias, documentation variability, and potential exposure misclassification. No structured dietary questionnaire or calibration protocol was applied during the chart abstraction process.

### 2.5. Statistical Analysis

Statistical analyses were performed using R software version 4.3.0 (R Core Team, 2023). Continuous variables are reported as means ± SD and categorical variables as frequencies (percentages). Caries increment (ΔDMFT) was compared between coffee consumers and non-consumers using independent samples t-tests, and across three consumption groups (non-consumers, sweetened coffee, and unsweetened coffee) using one-way ANOVA with Tukey’s HSD post hoc tests. Effect sizes were calculated post hoc as Hedges’ g for t-tests and eta-squared (η^2^) for ANOVA. A multivariable linear regression model was constructed to evaluate the association between coffee consumption and DMFT increment after adjusting for brushing frequency, sugary snack intake and OHI-S score. Shapiro–Wilk tests and Levene’s test were used to assess the normality of caries increment and the homogeneity of variance. A *p*-value of <0.05 was considered significant.

## 3. Results

### 3.1. Clinical Characteristics

A total of 375 adolescents were included (mean age 14.2 ± 1.3 years; 51.2% male). In total, 162 (43.2%) adolescents reported regular coffee consumption, and 213 (56.8%) reported no coffee intake. Baseline caries experience was comparable between groups: mean DMFT was 2.84 ± 1.25 in coffee consumers (95% CI, 2.65–3.03; n = 162) versus 2.97 ± 1.41 in non-consumers (95% CI, 2.78–3.16; n = 213), with a mean difference of −0.13 (95% CI, −0.40 to 0.14; *p* = 0.42). The mean follow-up duration was 4.12 ± 0.94 years (range: 1.8–5.0 years) for coffee consumers and 3.98 ± 1.05 years (range: 1.5–5.0 years) for non-consumers. The mean difference in follow-up duration between groups was 0.14 years (95% CI: −0.07 to 0.35; *p* = 0.192); see Table 1.

### 3.2. Primary Outcome

Coffee consumers exhibited a significantly lower mean caries increment than non-consumers over the study period: 0.78 ± 0.65 (95% CI: 0.68–0.88; n = 162) versus 1.34 ± 0.88 (95% CI: 1.22–1.46; n = 213), representing an absolute mean difference of −0.56 (95% CI: −0.71 to −0.41; *p* < 0.001) and a standardized mean difference (Hedges’ g) of −0.71 (95% CI: −0.92 to −0.50). This corresponds to an approximately 42% lower caries increment among coffee consumers compared to non-consumers.

One-way ANOVA comparing the three coffee consumption groups (non-consumers, sweetened coffee consumers, and unsweetened coffee consumers) revealed significant differences in mean ΔDMFT (F (2, 372) = 32.95, *p* < 0.001, η^2^ = 0.15). Post hoc Tukey’s HSD tests demonstrated that unsweetened coffee consumers had the lowest caries increment (0.52 ± 0.48; 95% CI: 0.42–0.62), which was significantly lower than both sweetened coffee consumers (1.09 ± 0.71; 95% CI: 0.94–1.24; mean difference = −0.57, 95% CI: −0.76 to −0.38, *p* < 0.001) and non-consumers (mean difference = −0.82, 95% CI: −0.98 to −0.66, *p* < 0.001). Sweetened coffee consumers also exhibited significantly lower caries increment than non-consumers (mean difference = −0.25, 95% CI: −0.44 to −0.06, *p* = 0.006). In relative terms, unsweetened and sweetened coffee consumption were associated with approximately 61% and 19% reductions in caries increment, respectively, compared to non-consumption; see Table 2.

### 3.3. Sensitivity Analysis

To address potential confounding by oral hygiene practices and dietary behaviors, we conducted multivariable linear regression analyses with caries increment (ΔDMFT) as the dependent variable. The model included coffee consumption category as the primary exposure variable and adjusted for three key confounders: tooth brushing frequency (times per day), consumption of sugary snacks and beverages (servings per week), and baseline oral hygiene status as measured by the Simplified Oral Hygiene Index (OHI-S). After controlling for these variables, habitual coffee consumption remained independently and significantly associated with lower caries increment. The adjusted mean difference in ΔDMFT compared to non-consumers was −0.22 (95% CI: −0.41 to −0.04; *p* = 0.019) for sweetened coffee consumers and −0.51 (95% CI: −0.70 to −0.33; *p* < 0.001) for unsweetened coffee consumers. Furthermore, direct comparison between coffee consumption types revealed that unsweetened coffee consumers experienced a significantly lower adjusted caries increment than sweetened coffee consumers (adjusted mean difference: −0.29, 95% CI: −0.51 to −0.07; *p* = 0.012). All adjustment variables demonstrated expected independent associations with caries increment in the multivariable model: tooth brushing frequency (β = −0.48, *p* < 0.001), frequency of sugary snacks/beverages (β = 0.14, *p* < 0.001), and OHI-S score (β = 0.35, *p* < 0.001).

## 4. Discussion

This retrospective cohort study found that adolescents who consumed coffee at least twice weekly had a significantly lower increment in DMFT compared with non-consumers, with the most pronounced benefit among those drinking coffee without added sugar. These findings align with a growing body of mechanistic and preclinical evidence indicating that bioactive constituents of coffee can interfere with the cariogenic process, primarily by suppressing *Streptococcus mutans* growth, adhesion, and biofilm formation, and by attenuating acidogenicity.

Polyphenol-rich beverages have long been posited as adjuncts to caries prevention. In vitro and ex vivo work shows that coffee and its principal compounds inhibit *S. mutans* growth and biofilms and can disrupt key virulence factors, including glucan-mediated adhesion to enamel. Almeida et al. demonstrated antimicrobial effects of caffeine, trigonelline, caffeic acid, protocatechuic acid, and chlorogenic acid against *S. mutans*, supporting a multi-compound basis for activity [15]. Ferrazzano et al. further reported that trigonelline, caffeine, and chlorogenic acid interfere with *S. mutans* adsorption to saliva-coated hydroxyapatite, suggesting reduced initial colonization and plaque maturation [12]. Comparative laboratory studies have also found antibacterial activity of coffee extracts relative to common oral antiseptics, though clinical translation remains to be established [16]. Importantly, the present study did not include microbiological assessment of *Streptococcus mutans* levels, salivary flow rate, salivary pH, buffering capacity, or direct plaque quantification. Therefore, while laboratory data provide biological plausibility, the current findings cannot establish a direct antimicrobial or mechanistic effect of coffee within this cohort. Any proposed biological pathway remains speculative and should be interpreted cautiously. Collectively, these data provide biologically plausible mechanisms that could explain the lower caries increment we observed among adolescent coffee consumers. However, whether oral exposure concentrations achieved through habitual coffee drinking reach the bacteriostatic thresholds demonstrated in controlled laboratory conditions remains uncertain.

Caries prevalence among children and adolescents in Saudi Arabia remains high, with recent syntheses reporting elevated rates and DMFT scores relative to international targets [17]. Large national and regional surveys continue to identify substantial disease burden in school-aged populations, despite preventive messaging [18]. Against this backdrop, culturally embedded, low-cost behaviors that can be modified at scale, such as the way coffee is prepared and consumed, may represent potential behavioral factors worthy of further investigation within preventive frameworks. Encouraging adolescents and families who already drink coffee to prefer unsweetened preparations may offer a feasible harm-reduction approach compatible with local customs.

Overall, these results suggest three practical considerations for clinicians. Dietary counseling may benefit from differentiating between coffee types; unsweetened coffee may represent a reasonable discussion point for adolescents and caregivers who already consume coffee, while emphasizing that added sugar may attenuate any potential benefits. Coffee should be framed as a possible adjunct rather than a replacement for evidence-based caries prevention, representing one additional consideration within a broader preventive plan. Given coffee’s acidity and potential for extrinsic staining, patients may be advised to consume it with meals, avoid frequent sipping between meals, and rinse with water afterward to limit erosive potential and pigment retention. Although the observed coffee consumption is modest, clinicians should remain mindful of total caffeine exposure in adolescents. Excessive intake may cause anxiety and sleep disturbance. Patients should be advised to limit overall caffeinated beverages and prefer unsweetened preparations.

Baseline oral hygiene differed between groups, with unsweetened coffee consumers demonstrating the most favorable practices. However, multivariable regression adjusting for tooth brushing frequency, OHI-S score, and dietary factors showed coffee consumption remained independently associated with reduced caries increment, suggesting oral hygiene differences do not fully explain the protective effect. The stronger association with unsweetened versus sweetened coffee supports a direct antimicrobial mechanism rather than purely behavioral confounding.

Nonetheless, several limitations warrant caution in interpreting these findings. Coffee intake and sweetening were abstracted from routine dietary histories and self-reported information and may be imprecise and subject to recall bias. Frequency does not capture dose, brew type, roast level, or preparation method, all of which can alter polyphenol profiles and oral effects. Although charts for key behaviors and basic health history were reviewed, unmeasured confounders such as socioeconomic status, parental education, health-seeking behavior, dietary discipline, and patterns of dental service utilization may partially explain the observed associations. Adolescents who habitually consume coffee—particularly unsweetened—may differ systematically in lifestyle characteristics not fully captured in clinical records. Residual confounding, therefore, cannot be excluded. Although fluoride exposure was screened as part of the eligibility criteria, granular data on fluoride toothpaste concentration and community water fluoridation levels were unavailable.

DMFT scores were recorded by routine attending clinicians rather than calibrated research examiners, and no inter- or intra-examiner reliability data were available for the chart abstraction period, which introduces a potential source of measurement heterogeneity that cannot be quantified retrospectively. Also, the use of a convenience sample from a single tertiary dental clinic limits the generalizability to adolescents in the broader community or those with less access to dental care. Although coffee consumption was documented at both baseline and follow-up, intermediate changes in consumption habits during the observation period were not ascertained. Adolescents who altered their frequency, preparation method, or quantity of intake between assessments would have been misclassified according to their terminal exposure status, introducing the possibility of non-differential exposure misclassification, which is inherent to this retrospective design. Furthermore, exposure classification was based on documentation at baseline and most recent follow-up rather than continuous monitoring across the observation period. Changes in coffee consumption patterns, preparation method, or sugar content between visits could have resulted in non-differential misclassification, potentially biasing estimates toward or away from the null. This limitation is inherent to retrospective chart-based designs.

Furthermore, participants in this study predominantly consumed traditional Arabic coffee (qahwa arabiyya), which is typically prepared using lightly roasted Arabica beans (*Coffea arabica*), coarsely ground and brewed with cardamom (*Elettaria cardamomum*) at varying concentrations depending on regional and individual preferences. Additional spices such as saffron, cloves, or cinnamon may occasionally be incorporated, though cardamom remains the most ubiquitous additive. Cardamom essential oils have demonstrated bacteriostatic and bactericidal activity against *Streptococcus mutans* in controlled laboratory conditions, and may contribute to biofilm disruption through interference with bacterial quorum sensing [19,20]. Consequently, the observed caries-protective association may reflect the combined effects of coffee polyphenols and cardamom-derived bioactive compounds rather than coffee alone. These findings should not be interpreted as a recommendation to initiate coffee consumption for caries prevention.

## 5. Conclusions

Within the constraints of a retrospective exploratory design, habitual coffee consumption, particularly unsweetened, was associated with lower caries increment among adolescents attending a university dental clinic. However, the reliance on non-standardized dietary documentation, the absence of biological measurements, and the potential for residual confounding limit causal interpretation. The findings should therefore be considered hypothesis-generating. Prospective longitudinal studies incorporating validated dietary assessment tools, calibrated examiners, microbiological analyses, and salivary parameters are required to clarify whether coffee exerts a true anticariogenic effect or reflects underlying behavioral patterns.

## Figures and Tables

**Table 1:** Baseline characteristics of participants.

Characteristic	Non-Coffee Consumers	Sweetened Coffee Consumers	Unsweetened Coffee Consumers	*p*-Value
Age (years), mean ± SD	14.1 ± 1.4	14.3 ± 1.2	14.3 ± 1.2	0.348
Male	110 (51.6%)	40 (49.4%)	42 (51.9%)	0.934
Baseline DMFT, mean ± SD (95% CI)	2.97 ± 1.41 (2.78–3.16)	2.88 ± 1.28 (2.60–3.16)	2.80 ± 1.22 (2.53–3.07)	0.420
Tooth Brushing Frequency (times/day), mean ± SD	2.0 ± 0.5	2.1 ± 0.4	2.2 ± 0.5	0.005
Sugary Snacks/Drinks (servings/week), mean ± SD	4.7 ± 1.9	4.6 ± 1.7	4.2 ± 1.6	0.103
Regular Fluoride Exposure, n (%)	198 (93.0%)	75 (92.6%)	77 (95.1%)	0.775
Oral Hygiene Score (OHI-S Index), mean ± SD	1.3 ± 0.7	1.2 ± 0.5	1.1 ± 0.5	0.042

**Table 2:** Summary of outcomes in sub-groups.

Outcome Measure	Non-Coffee Consumers (N = 213)	Sweetened Coffee Consumers (N = 81)	Unsweetened Coffee Consumers (N = 81)
ΔDMFT Increment, mean ± SD (95% CI)	1.34 ± 0.88 (1.22–1.46)	1.09 ± 0.71 (0.94–1.24)	0.52 ± 0.48 (0.42–0.62)
Number of New Cavitated Lesions, mean ± SD	1.5 ± 1.0	1.2 ± 0.8	0.6 ± 0.5
Progression to Missing/Filled Teeth, n (%)	75 (35.2%)	25 (30.9%)	12 (14.8%)

## Data Availability

The data supporting this study’s results can be obtained from the corresponding author upon reasonable request. Individual patient data are not publicly available to ensure patient privacy.
